# FRZB: a potential prognostic marker for head and neck squamous cell carcinoma

**DOI:** 10.1590/1414-431X2024e13368

**Published:** 2024-05-17

**Authors:** Yunshan Li, Feihan Gu, Xu Huang, Wenkai Huang, Junwei Xiang, Jiayuan Yue, Yuanyin Wang, Ran Chen

**Affiliations:** 1 College & Hospital of Stomatology, Anhui Medical University, Key Laboratory of Oral Diseases Research of Anhui Province, Hefei, China

**Keywords:** FRZB, HNSCC, Prognostic marker, Immune infiltration, TCGA

## Abstract

Head and neck squamous cell carcinoma (HNSCC) is the sixth most common malignancy worldwide, with approximately 600,000 new cases each year. A small number of HNSCCs are caused by human papillomavirus (HPV) infection. Frizzled related protein (FRZB) has been reported in many inflammatory diseases and cancers, but it is yet unclear how FRZB affects HNSCC, as well as its role and underlying mechanism. TIMER2 database was utilized to evaluate FRZB expression in cancer tissues, and FRZB expression in HNSCC tissues was confirmed by samples obtained from Gene Expression Omnibus. To identify whether FRZB could be used as a prognostic predictor, we performed univariate and multivariate Cox regression analyses. FRZB co-expression profile was explored using the LinkedOmics database, then Kyoto Encyclopedia of Genes and Genomes and Gene Ontology enrichment analyses were performed for these FRZB-related genes in HNSCC samples. Lasso regression analysis was subsequently used to screen for prognostic variables, and we determined the infiltration of immune cells in HNSCC patients to clarify the influence of FRZB on tumor immune microenvironment. At last, we assessed the association between FRZB expression and immune checkpoint gene, and compared the sensitivity of common chemotherapeutic agents. In this study, we found that FRZB was dysregulated in HNSCC tumor tissues and had a relationship with clinical parameters. The reliability and independence of FRZB as a factor in determining a patient's prognosis for HNSCC was also established. Additional investigation revealed that FRZB was linked to common immune checkpoint genes and may be implicated in immune infiltration.

## Introduction

Most of the head and neck cancers, known as head and neck squamous cell carcinoma (HNSCC), originate in the mucosal epithelium of the larynx, pharynx, and oral cavity (1). Alcohol abuse, tobacco consumption, or both are commonly linked to larynx and oral cavity cancers. On the other hand, human papillomavirus (HPV) infection, especially HPV-16, is increasingly confirmed as the cause for pharynx cancers (1). As a result, HNSCC could be classified as HPV-positive or HPV-negative HNSCC. Despite signs of a histological transition from cellular atypia to varying degrees of dysplasia, which ultimately results in invasive HNSCC, without a preceding pre-malignant lesion that is clinically evident, most patients are diagnosed with late-stage HNSCC (2), greatly increasing the difficulty of clinical treatment.

Surgical resection is generally the first choice for HNSCC of the oral cavity. Depending on the disease stage, chemotherapy plus radiotherapy or adjuvant radiotherapy (known as CRT or chemoradiation) may then be given (3). CRT has been the most common treatment of cancers that occur in the larynx or pharynx. Compared to HPV-negative HNSCCs, HPV-positive HNSCCs have a more favorable prognosis, and in the treatment of HPV-positive cancers, the effectiveness of therapeutic dose reduction is being tested in ongoing studies (4). Except for larynx cancers or early-stage oral cavity cancers, multidisciplinary care and multimodality approaches are required for the treatment of HNSCC cases. Immune profiling of HNSCC as well as detailed molecular characterization allow targeted therapies to be more effective by incorporating predictive and prognostic biomarkers into clinical management (5), thereby prolonging survival. Molecular biomarkers, therefore, have become one of the hot spots in tumor treatment.

Known as sFlRP3, frizzled related protein (FRZB) is from the family of secreted Fz-related proteins, and as a member of this family, it has the characteristic of having a cysteine-rich domain (CRD) with Fz receptors. By linking to extracellular Wnt ligands, FRZB blocks receptor signaling and then prevents ligand-receptor interaction (6). FRZB has been reported in many inflammatory diseases, especially osteoarthritis. For instance, compared to wild-type control mice, transcriptome analysis of subchondral bone and articular cartilage indicates that FRZB−/− mice have cell cycle, cell adhesion, and extracellular matrix alterations. This may lead to FRZB−/− mice being more susceptible to experimentally-induced osteoarthritis (7). FRZB has been reported in cancer as well. According to Guo et al. (8), as the secreted Wnt antagonist, FRZB can decrease invasiveness and growth of fibrosarcoma cells linked to inhibition of Met signaling. In gastric cancer, FRZB could suppress cell proliferation and modulate the balance between differentiation and proliferation (9). FRZB was low-expressed in triple-negative breast cancer (TNBC) and was shown to be regulated by EGR1, via modulation of the JAK/STAT3 pathway, and thus inhibit growth and invasion of TNBC cell (10). These results suggest that FRZB may play a critical role in malignant tumors, but it is yet unclear how FRZB affects HNSCC, as well as its role and underlying mechanism. Therefore, we hypothesized that FRZB is a potential biomarker of HNSCC and plays a role in its treatment.

In this study, we investigated whether FRZB has an influence on immune infiltration in HNSCC and its predictive value by using extensive bioinformatics analysis; the workflow of this study is presented in Supplementary Figure S1. We contrasted FRZB expression between tumor and normal tissues, then evaluated the association between FRZB expression and the clinical features of HNSCC patients. We evaluated the prognostic role of FRZB and established the FRZB-related risk model. By using multiple algorithms, we comprehensively analyzed the immune infiltration landscape.

## Material and Methods

### Data collection and process

The sequence data and the relative clinical information was collected from The Cancer Genome Atlas (TCGA). As the largest cancer genetic information database for large-scale genome sequencing and other data like proteomic, epigenetic, transcriptomic, and genomic, TCGA database includes 33 types of cancer. As Supplementary Table S1 shows, we found the expression of FRZB and complete clinical data for 499 HNSCC patients. TIMER2 webserver was employed for analyzing FRZB expression of pan-cancer (11). In order to determine the expression of FRZB in HNSCC patients, the datasets GSE30784, GSE25099, and GSE37991 were collected from the Gene Expression Omnibus (GEO). GSE30784 includes 167 oral squamous cell carcinoma (OSCC) samples and 45 adjacent normal samples from the GPL570 platform [HG-U133_Plus_2] Affymetrix Human Genome U133 Plus 2.0 Array. The GPL5175 platform [HuEx-1_0-st] Affymetrix Human Exon 1.0 ST Array was used to obtain GSE25099, which includes 22 adjacent normal samples and 57 OSCC samples. GSE37991 was from GPL6883 Illumina HumanRef-8 v3.0 expression bead chip, and includes 40 OSCC and 40 adjacent normal samples. FRZB expression was analyzed in different clinical subgroups comprehensively by using UALCAN webserver (12).

### Evaluation of the prognostic value

Kaplan-Meier survival curves of FRZB were created by the Kaplan-Meier plotter (13). To identify whether FRZB could be used as a prognostic predictor, univariate and multivariate Cox regression analyses were performed.

### Analysis of FRZB co-expression and functional enrichment

For co-expression analysis of FRZB, we used the HNSCC cohort from TCGA (14). Genes with adjusted false discovery rate (FDR) <0.05 and |cor| >0.4 were used as standard for co-expressed genes in the Pearson correlation test on the LinkOmics Portal (15) (Supplementary Table S2). Based on the median expression value of FRZB, HNSCC samples from TCGA were divided into two groups: those with low expression and those with high expression. We identified the genes that were differentially expressed between the two groups by the Limma package in R with standard FDR <0.05 and |logFC| >1.0. The genes that were correlated with FRZB were analyzed by Gene Ontology (GO) and Kyoto Encyclopedia of Genes and Genomes (KEGG) enrichment with the R package “clusterProfiler” (16).

### Construction of risk assessment model

We employed univariate cox regression analysis to assess associations among genes and prognosis in patients with HNSCC in order to identify survival-related FRZB genes. For further analysis, we choose genes with P-values less than 0.05 as candidates. To avoid overfitting, we used LASSO regression analysis to obtain the best prognostic genes. Then, multivariate Cox regression analysis was used to build an optimized risk score. The HNSCC suffers' risk score was calculated as follows: $\text{Risk score}=\sum_{i}^{7}Xi\times Yi$ (where Yi is gene expression and Xi is the risk factor). ROC curves were created at 1, 3, and 5 years to identify cutoff points for low- or high-risk scores, at every point of the 5-year ROC curve, and the values were then evaluated by the Acak Information Criterion (AIC). To evaluate the accuracy of this cutoff and show the difference in survival rates between the two groups, Kaplan-Meier survival analysis was used. The survival curves and risk scores for each subject were created using R tools. We assessed the model's capacity for prediction with the R packages “survival”, “survival ROC”, and “survminer”.

### Immune infiltration analysis

Established methods, like QUANTISEQ, TIMER, XCELL, and other methods, were utilized to evaluate the relationship between FRZB expression and immune cell infiltration and the degree of immune infiltration in HNSCC patients (17-22). Each HNSCC sample's tumor immune microenvironment (TME) status was also assessed utilizing the ESTIMATE package in R, and immune/stromal/ESTIMATE scores were displayed for the results (23). High expression and low expression group scores were contrasted using the ggpubr package in R. To find changes in immune function between these groups, single-sample Gene Set Enrichment Analysis (ssGSEA) was also used. In order to identify immunological pathways, we also employed the GSVA package in R, which was enriched in the low and high FRZB expression groups.

### Analysis of chemotherapeutic sensitivity and immune checkpoint gene

It is known that immune checkpoint inhibitors play a critical role in immunotherapy. Using TIMER2, we assessed the relationship between FRZB expression and immune checkpoint genes. Based on the anticancer drug sensitivity data acquired from the Genomics of Drug Sensitivity in Cancer (GDSC), we utilized the R package “pRRophetic” to calculate the half-maximal inhibitory concentration (IC50) of chemotherapeutic drugs in HNSCC suffers to assess the influence of FRZB on the treatment of HNSCC (24,25). Via the Wilcoxon rank-sum test, we compared the chemotherapeutic sensitivity of the high and low FRZB expression groups, and box plots were used to visualize the results. In addition, the relationship between the expression of FRZB and treatment responsiveness was examined using the CellMiner NCI-60 cancer cell line (26).

### Statistical analysis

The GEO database was used to retrieve information about FRZB expression levels. For the Cox regression analysis, both univariate and multivariate analyses were used. The Wilcoxon rank-sum test was used to analyze the differences in IC50 values, immune checkpoint genes expression, and TME scores. Pearson correlation analysis was used to estimate the relationship between immune infiltration cell scores and FRZB expression. The 4.1.1 version of the R software (R Core Team) was used for all statistical analyses. Unless otherwise stated, P<0.05 was used to indicate statistically significant results.

## Results

### Low expression of FRZB in HNSCC

**Figure 1:**
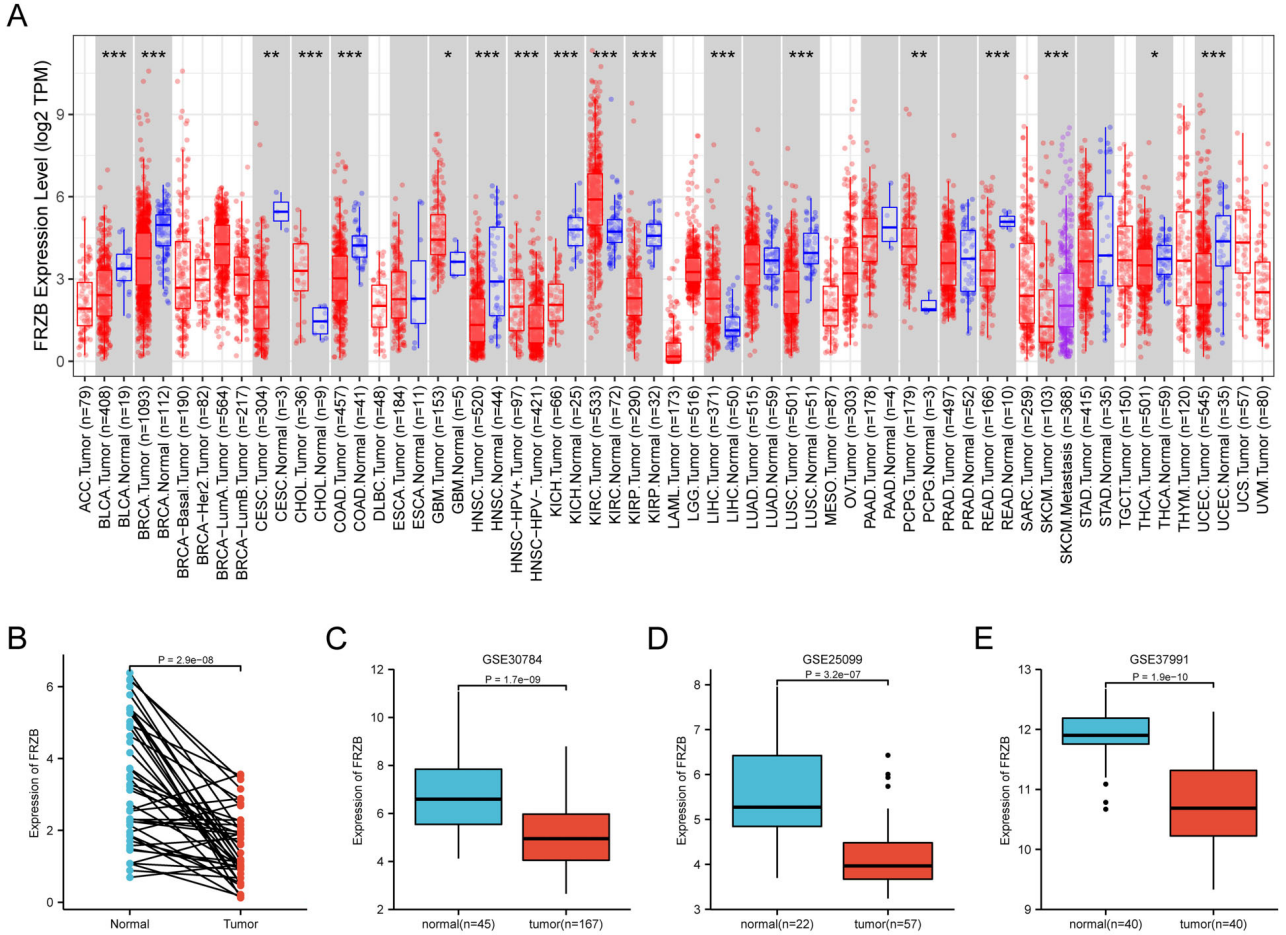
Frizzled related protein (FRZB) in head and neck squamous cell carcinoma (HNSCC). **A**, FRZB expression in pan-cancer. **B**, The expression of FRZB was decreased in tumor tissues compared with normal tissues. **C**-**E**, Lower expression of FRZB in HNSCC tissues was confirmed by samples obtained in different datasets from Gene Expression Omnibus database. Data are reported as median and interquartile range (Wilcoxon test).

Results showed that FRZB expression was lower in tumors than in the corresponding normal tissues in BRCA, HNSC, BLCA, COAD, CESC, LUSC, KICH, KIRP, READ, UCEC, and THCA. In contrast, FRZB expression was higher in GBM, CHOL, LIHC, PCPG, and KIRC (Figure 1A). In order to further verify whether FRZB expression level in HNSCC was lower, we used TCGA datasets. From the obtained results, the expression of FRZB was decreased in tumor tissues compared with adjacent normal tissues (Figure 1B). Lower expression of FRZB in OSCC tissues was confirmed by samples obtained from GEO (accession numbers: GSE37991, GSE30784, and GSE25099) (Figure 1C-E).

**Figure 2:**
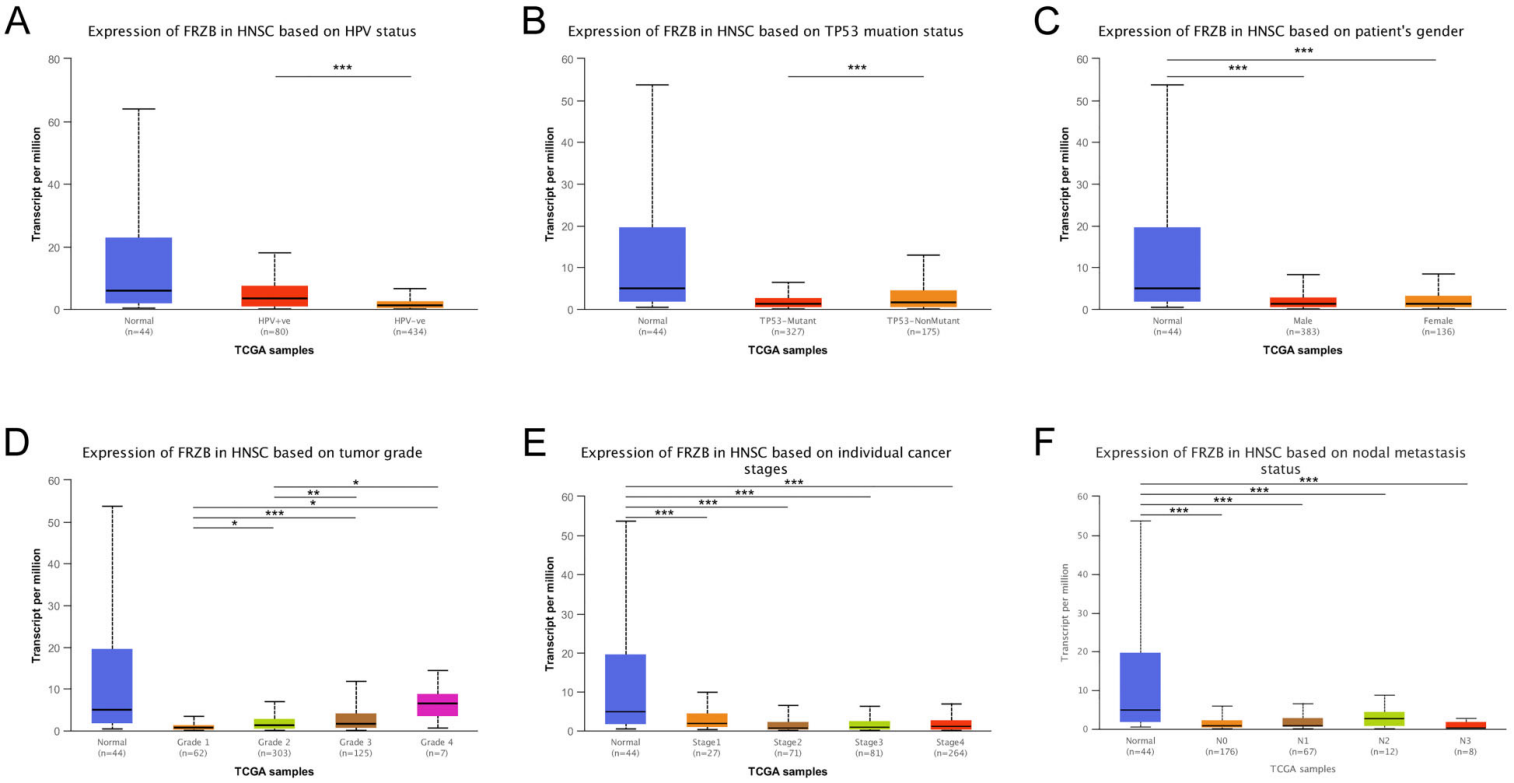
**A-F**, Frizzled related protein (FRZB) expression in different subgroups of head and neck squamous cell carcinoma. Data are reported as median and interquartile range. *P<0.05, **P<0.01, ***P<0.001; Kruskal-Wallis test.

The UNCLAN software (England) was used to evaluate FRZB expression differences between HNSCC clinical subgroups and normal samples. As shown in Figure 2A-F, FRZB expression was significantly down-regulated in different subgroups of HNSCC patients, including TP53 mutation status, HPV status, metastasis,gender, and tumor grade and stage, indicating that FRZB may be a potential biomarker for patients with HNSCC.

### Prognostic value of FRZB in HNSCC

**Figure 3:**
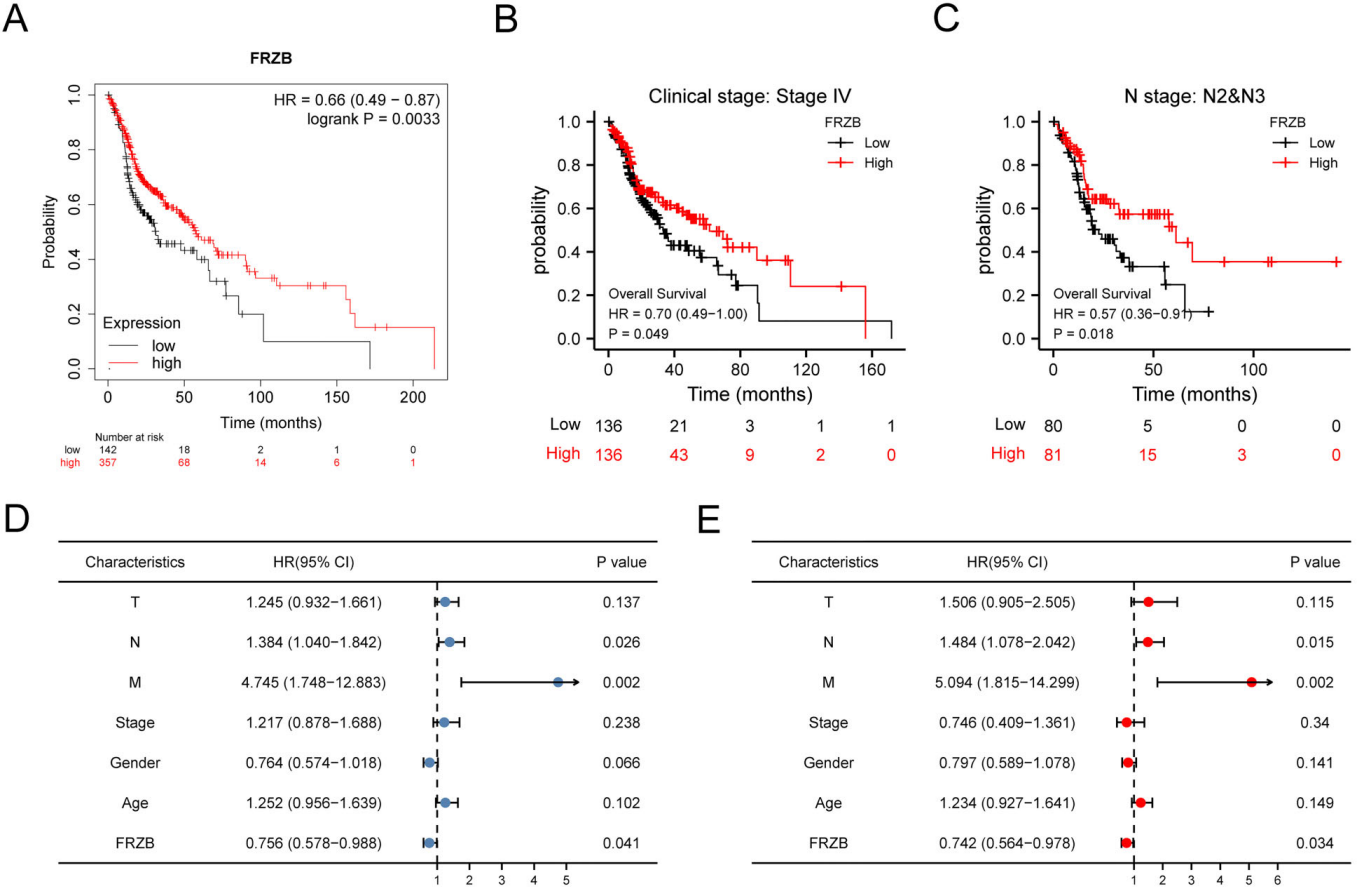
The head and neck squamous cell carcinomas (HNSCC) with high expression of frizzled related protein (FRZB) had a better prognosis. **A**, Overall survival curves by Kaplan-Meier. **B** and **C**, Survival curves in advanced HNSCC patients. **D** and **E**, Univariate and multivariate analysis results showing the hazard ratio (HR) of several factors related to better survival in samples from The Cancer Genome Atlas (TCGA). T: tumor; N: node; M: metastasis.

Kaplan-Meier plots indicated that HNSCC patients with higher expression of FRZB have a better prognosis (Figure 3A). The results revealed that FRZB was an important protection factor of patients with advanced HNSCC (Figure 3B and C). Univariate Cox regression analysis showed that FRZB was closely related to overall survival, and multivariate regression analysis further indicated that FRZB may be an independent prognostic factor for HNSCC patients (Figure 3D and E).

### Analysis of FRZB-related genes

**Figure 4:**
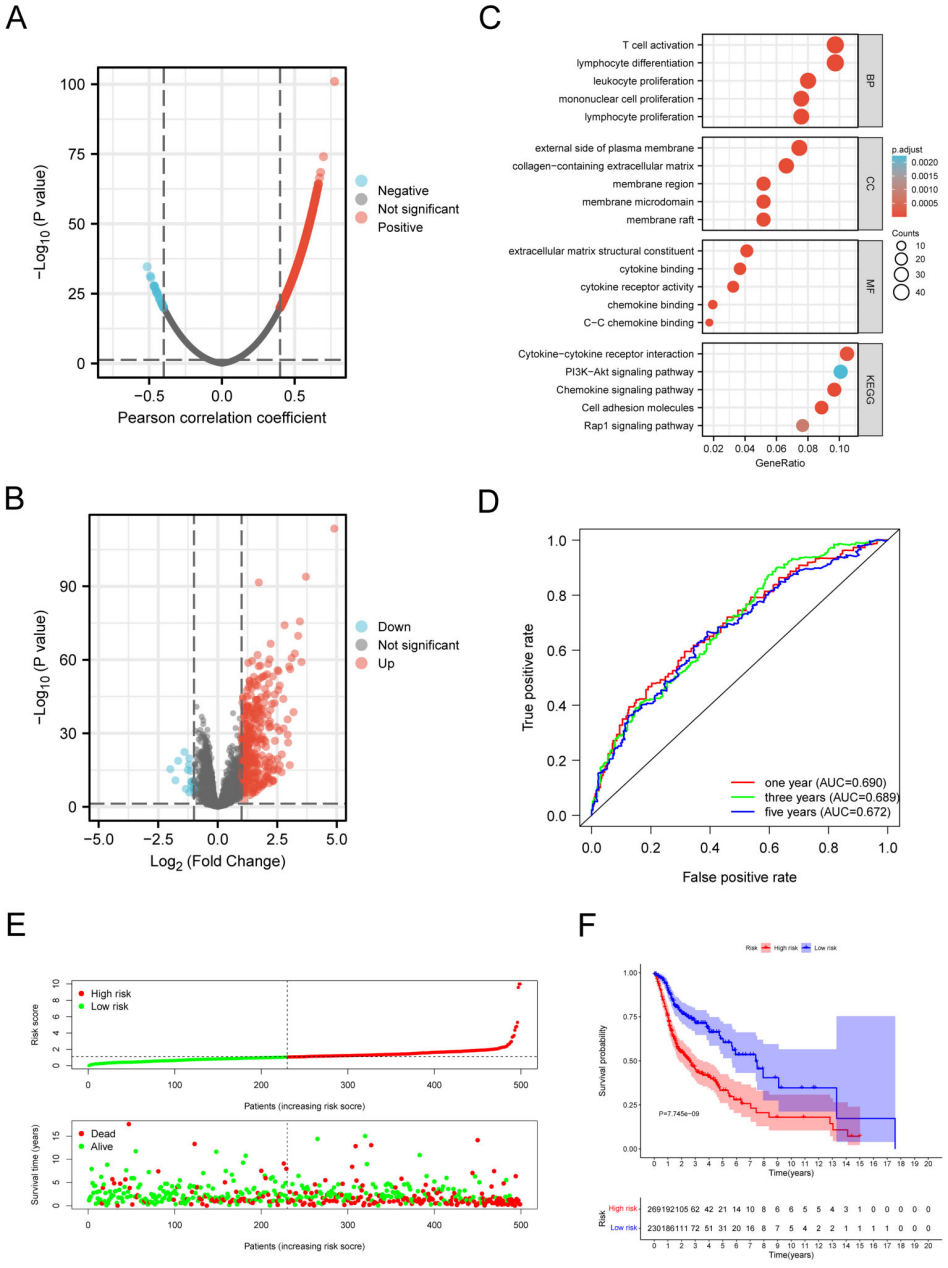
Functional enrichment and risk model. **A** and **B**, Frizzled related protein (FRZB) co-expression profile was explored in head and neck squamous cell carcinoma tissues using the LinkedOmics database. **C**, Gene Ontology (GO) and Kyoto Encyclopedia of Genes and Genomes (KEGG) enrichment analyses. BP: biological process; CC: cell components; MF: molecular functions. **D**, The model of risk assessment showed good sensitivity. **E**, Risk scores and (**F**) survival of low-and high-risk groups.

FRZB co-expression profile in HNSCC was explored using the LinkedOmics database, and 1483 genes were found (|cor| >0.4, FDR <0.05) (Figure 4A). Between the low expression groups and high expression groups, 1969 genes were found to be differentially expressed (Figure 4B), and after deletion of duplicate genes, 2768 FRZB-related genes were obtained. Then KEGG and GO enrichment analyses were performed for these FRZB-related genes in HNSCC samples. In the category of biological process (BP), the main enrichment of these differentially expressed genes was in T cell activation and lymphoid cell differentiation, and in the category of cell components (CC), these genes mainly occurred in the external side of the plasma membrane. On the other hand, the molecular functions (MF) enrichment results showed that these genes were mainly related to the extracellular matrix structure composition, cytokines, and cell factor receptor activity. In addition, the enrichment of these differentially expressed genes of KEGG pathway analysis revealed that the main pathways were the PI3K-Akt signaling pathway and chemokine signaling pathway and cytokines-cell factor receptor interaction (Figure 4C). These findings suggested that FRZBS may take part in the immune response in HNSCC and could have an influence on the efficacy of immunotherapy through multiple mechanisms.

### Establishment and evaluation of FRZB-related risk model

In this study, we initially identified 214 survival-related FRZB genes (Supplementary Table S3). Lasso regression analysis was subsequently used to screen for prognostic variables, and 16 FRZB-related genes were identified (Supplementary Figure S2A and B). Eight prognosis-related genes were finally used to establish the HNSCC risk assessment model (Supplementary Figure S2D and E). In 1-year, 3-year, and 5-year survival rate curves, the areas were 0.678, 0.721, and 0.668, indicating that the model had enough sensitivity for predicting survival (Figure 4D). In addition, we used a cutoff value of 1.054 to divide patients into low-risk and high-risk groups, with 269 patients in the high-risk group and the remaining 230 patients in the low-risk group (Supplementary Figure S2C). Figure 4E shows the risk scores and survival rates for each case, indicating that compared to patients in the high-risk group, the low-risk group patients could have better clinical outcomes. The Kaplan-Meier analysis and corresponding survival curves showed that, in contrast to patients with low-risk, the survival time of high-risk HNSCC patients was obviously shorter (P<0.001; Figure 4F).

### Relationship between FRZB and tumor microenvironment

**Figure 5:**
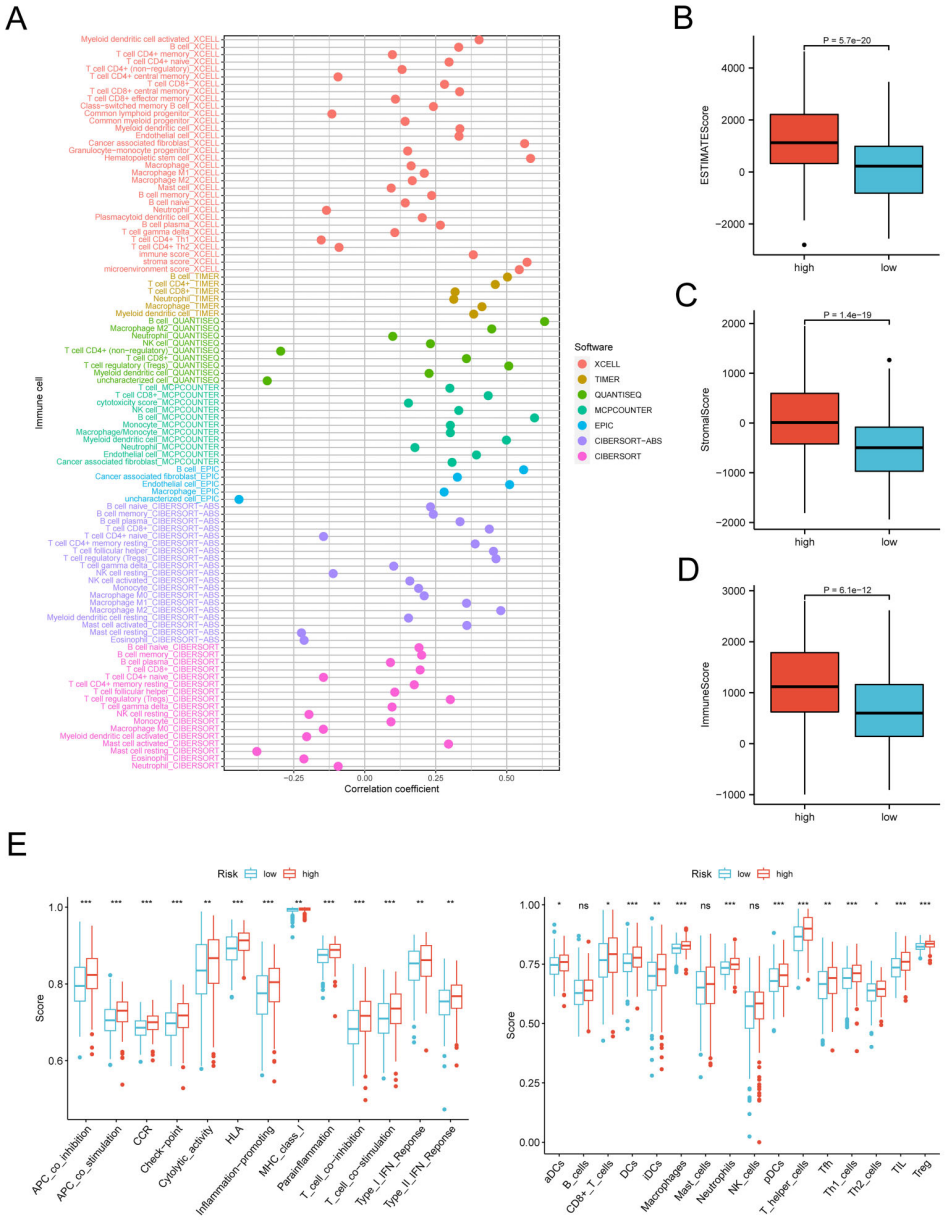
Estimation of immune-infiltrating cells. **A**, The frizzled related protein (FRZB) expression was related to immune cells in head and neck squamous cell carcinoma (HNSCC) samples. **B**, Estimated score, **C**, stromal score, and **D**, immune score of HNSCC of high and low FRZB expression. **E**, Immune response factors of high and low FRZB expression HNSCC patients. Data are reported as median and interquartile range. *P<0.05, **P<0.01, ***P<0.001; Wilcoxon test. ns: non-significant.

We determined the infiltration of immune cells in HNSCC patients to clarify the influence of FRZB on TME. The expression of FRZB was positively correlated with most immune cells, such as NK cells (r=0.332, P<0.001), B cells (r=0.503, P<0.001), regulatory T cells (Tregs) (r=0.302, P<0.001), CD8+ T cells (r=0.440, P<0.001), cancer-associated fibroblast (r=0.308, P<0.001), and CD4+ T cells (r=0.460, P<0.001) (Figure 5A, Supplementary Table S4). On the other hand, these results also showed that the stromal score, immune score, and estimated score were obviously lower in the FRZB low expression group than in the FRZB high expression group (Figure 5B-D). The results of ssGSEA further suggest that the high FRZB expression patients may have a more active immune response than those with low FRZB expression (Figure 5E).

### Prospects of FRZB in the treatment of HNSCC

**Figure 6:**
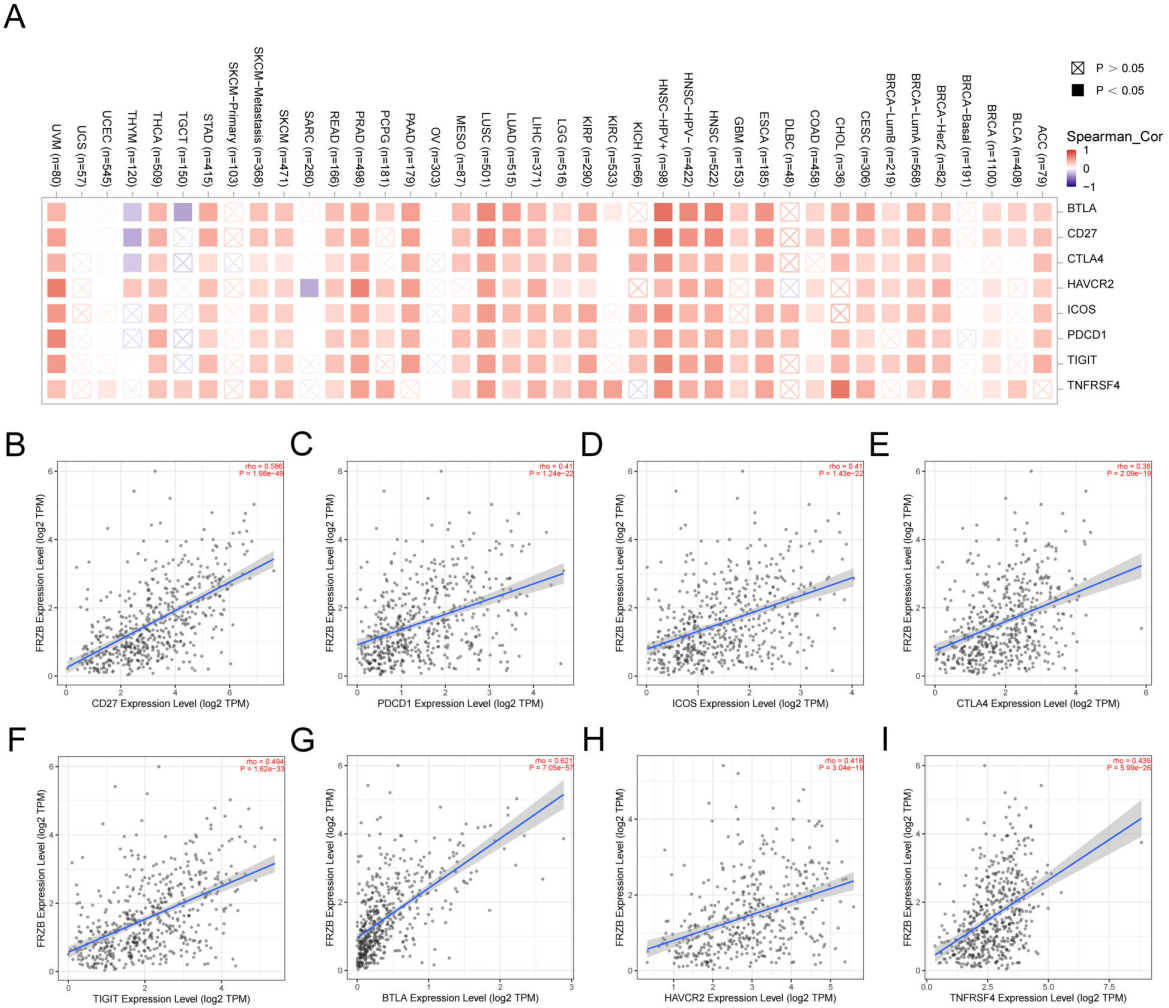
Correlation analysis between checkpoint-related genes and frizzled related protein (FRZB) expression in pan-cancer (**A**). The FRZB expression had a significant correlation to several immune checkpoints (**B**-**I**).

TIMER2 was used to detect the correlation between FRZB expression and the expression of major immune checkpoint genes including BTLA, CD27, CTLA4, ICOS, HAVCR2, TIGIT, PDCD1, and TNFRSF4. Figure 6A-I shows that FRZB expression in HNSCC was obviously related to the above immune checkpoint genes. It is interesting to note that in HPV-positive patients with HNSCC, FRZB expression and immunologic checkpoint gene expression had a stronger positive correlation, especially CD27 and BTLA. Analysis based on the GEO database also showed a strong association of FRZB with the above immune checkpoints (Supplementary Figure S3).

**Figure 7:**
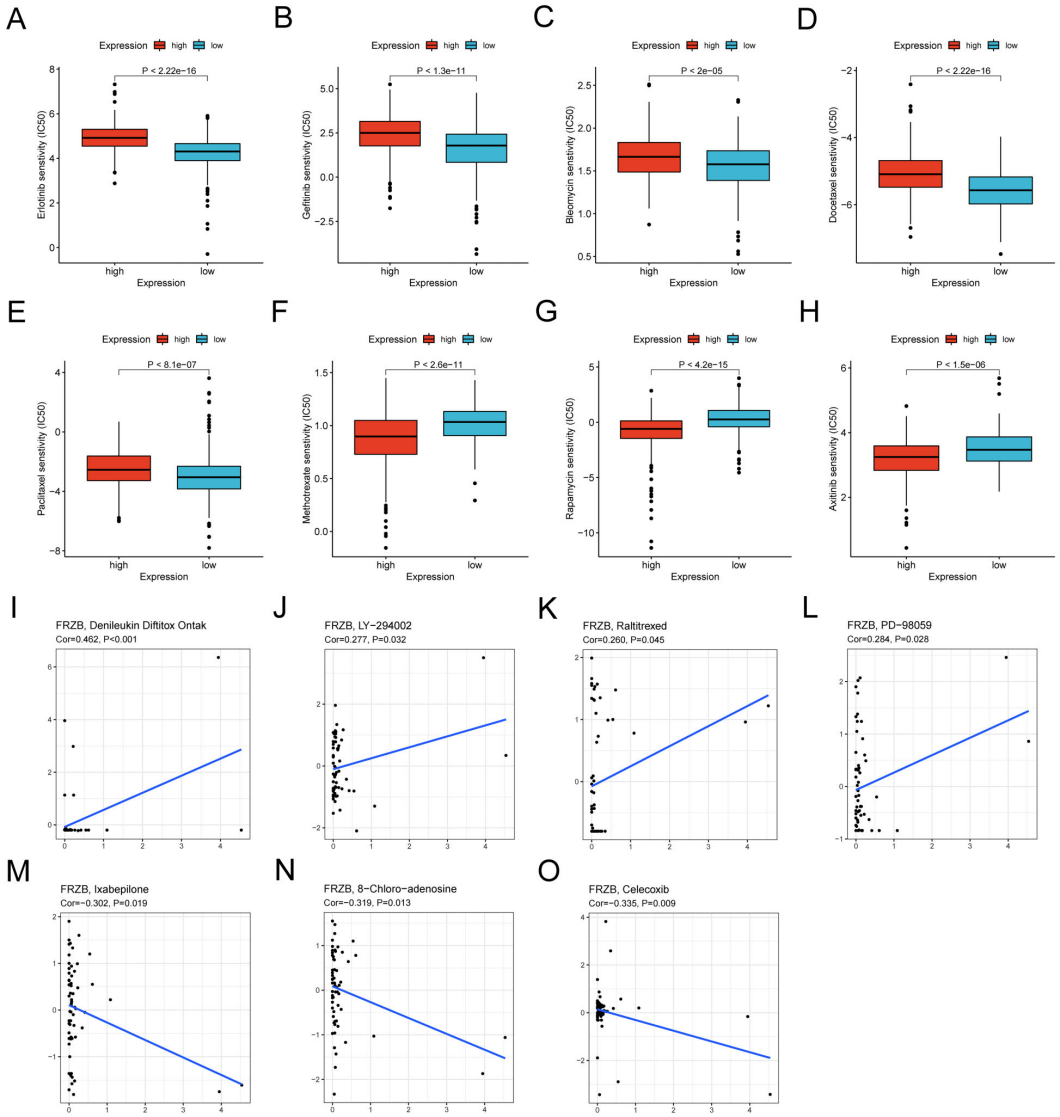
**A-H**, Chemotherapeutic sensitivity in high and low expression of frizzled related protein (FRZB) HNSCC subgroups. Data are reported as median and interquartile range. *P<0.05, **P<0.01, ***P<0.001; Kruskal-Wallis test. **I**-**O**, Correlation analysis between FRZB expression and anticancer drugs.

The pRophetic package in R was used to compare the sensitivity of common chemotherapeutic agents between FRZB high- and low-expression groups. According to the results, in patients with low FRZB expression, the IC50 values of erlotinib, gefitinib, bleomycin, docetaxel, and paclitaxel were lower (Figure 7A-E), and in contrast, the IC50 values of methotrexate, rapamycin, and axitinib were higher (Figure 7F-H). Data from CellMiner indicated that FRZB expression was related to the sensitivity to seven anticancer drugs. Four drugs for the treatment of patients with FRZB expression were positively related to drug response, including denileukin diftitox, LY-294002, raltitrexed, and PD-98059 (Figure 7I-L), but negatively correlated to the response to ixabepilone, 8-chloro-adenosine, and celecoxib (Figure 7M-O).

## Discussion

Many patients with HNSCC present with locally advanced disease and noticeable lymph node involvement (27). For locally advanced disease, conventional treatments consist of a combination of chemotherapy, radiotherapy, and surgery, which may lead to significant short or long term morbidity and provide a cure in around 50% of cases. Because of its metastatic nature, a large excision and neck lymph node dissection are usually required. In some patients, parts of the tongue, cheek, jaw, and other parts are removed, which not only results in great pain, and a poor prognosis, but also to different degrees of psychological problems for the patients.

In recent years, the emergence of biomarker-targeted therapy greatly reduced the pain, and improved the quality of life of patients (28). In this study, we found that FRZB is dysregulated in HNSCC tumor tissues, and has a relationship with clinical parameters. The reliability, and independence of FRZB as a prognostic factor in HNSCC was also established. In addition, FRZB was associated to common immune checkpoint genes and may be implicated in immune infiltration.

According to the results, we found that the expression of FRZB was obviously downregulated in multiple cancers including HNSCC compared to samples of non-tumor tissues. Three separate datasets from the GEO database were used to further confirm the differential FRZB expression in HNSCC. Patients with higher expression of FRZB would have superior survival rates, according to survival analysis. In addition, it was shown that FRZB can function as a stand-alone protective factor for HNSCC patients. For the purpose of determining the underlying mechanism and molecular roles of FRZB in HNSCC, GO, and KEGG enrichment analyses were employed to evaluate the FRZB-related genes. These findings showed that FRZB was related to HNSCC risk variables and may have a role in T cell activation. These results supported the hypothesis that in HNSCC, FRZB could actively contribute to tumor immune surveillance and defense. Moreover, the FRZB-related risk assessment model was ultimately developed, and it was shown that the model had a significant predictive value for evaluating the prognosis of HNSCC patients.

While environmental carcinogens like HPV infection, alcohol, or tobacco are directly linked to the development of HNSCC, defects in the immune response may have a critical role in cancer progression and establishment (29). Many studies showed that in patients with advanced HNSCC, immune cell dysfunction can be found in the TME and peripheral blood (30). Immunosuppressive effects in the TME may protect tumors from immune recognition and elimination, but may favor therapeutic intervention (31).

Diverse changes such as modulation of immune checkpoints, quantitative and qualitative changes in immune cell populations, TME factors like secretion of cytokines, together with a deficient antigen presenting machinery can disrupt the immune milieu balance of tumor cells, and may help the tumor escape immune surveillance (32). The expression of human leukocyte antigen (HLA) class 1 can be reduced or altered in the tumor cell. There are a variety of modifications in immune infiltrates in HNSCC tumors, ranging from the absence of immune cells to abundance of immune effector cells, like of NK or TILs cells, which are present but have poor function (33). These alterations may be accompanied by the presence of immunosuppressive cells together with tumor-associated macrophages and T regulatory cells. Within the TME, across diverse cell populations, immune checkpoints, such as LAG-3, TIM-3, CTLA-4, and PD-L1, were found upregulated (34). Tumor cells could produce immunosuppressive cytokines including TGF-β, IL-10, IL-6, VEGF, and GM-CSF, and local and systemic immunosuppressive effects may be exerted by immunosuppressive cell populations (35). In addition, immune cell trafficking and function and cytokine release may be influenced by TME factors such as high interstitial pressure, abnormal vasculature and lymphatics, and hypoxia. Incorporation of immunologic and genomic evaluation and immune TME comprehensive characterization across HNSCC can not only help regulate the immune system's healing potential but also provide prognostic information about tumor behaviors (36).

The immune landscape of the host and tumor can help predict how patients will benefit from immunotherapy (37). Importantly, the use of immune checkpoint inhibitors has shown long-lasting effects in a variety of cancers (38). Unfortunately, a significant fraction of immune checkpoint treatments has adverse effects on patients, the availability of prognostic biomarkers is constrained, and the response to such therapies is diverse and currently unknown (39). Research is now being done to identify and examine possible biomarkers that indicate a patient's response to immunotherapy. A better knowledge of immunity is expected to help harness the full potential of immunotherapy and enable patients to get suitable therapies. In addition to the discovery of novel biomarkers, the assays and platforms used to precisely and repeatedly assess biomarkers have an important influence on assuring consistency of measurement both within and across patients (40).

The current study has several limitations. First, neither *in vitro* nor *in vivo* studies of FRZB's influence on the malignant development of HNSCC cells were conducted. In light of this, it is necessary to confirm the results of this study in further research. Second, further information is needed to understand how FRZB expression and immune infiltration are regulated in HNSCC. Moreover, gene-based markers may not be enough as biometric features or as prognostic factors to predict patient outcomes. For forecasts to be more accurate and relevant, network or sub-network markers should be constructed.

### Conclusion

In summary, the current investigation showed that FRZB expression was low in HNSCC tissues, and high expression of FRZB was associated with increased infiltration of immune cells and a better prognosis for HNSCC patients. The results also emphasized FRZB's crucial function in its prognostic and therapeutic significance.

## Supplementary Material

Click here to view [zip].
