# Supplementary material for: FRZB: a potential prognostic marker for head and neck squamous cell carcinoma
Source: Braz J Med Biol Res. 2024 May 17;57:e13368. doi: 10.1590/1414-431X2024e13368 (PMC11101165; doi:10.1590/1414-431X2024e13368)

Figure S1. Flow diagram of the study. FRZB: frizzled related protein; HNSCC: head and neck squamous cell carcinoma; GEO: Gene Expression Omnibus; TCGA: The Cancer Genome Atlas.

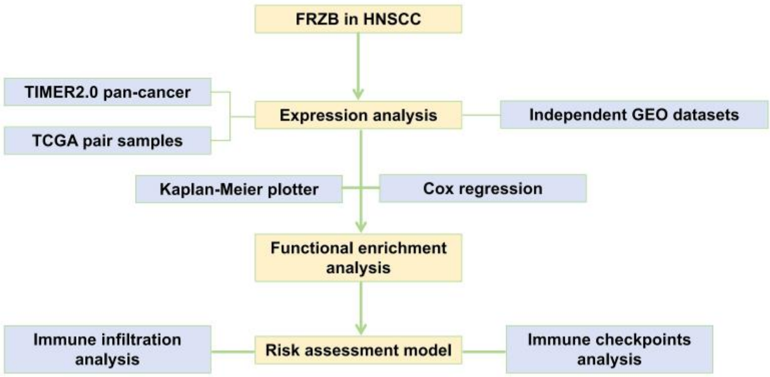

Figure S2. A and **B**, LASSO regression of frizzled related protein (FRZB)-related genes. **C**, The maximum inflection point on the 3-year receiver operating curve curve was 1.054. **D** and **E**, Eight prognosis-related genes that were used to establish the head and neck squamous cell carcinoma (HNSCC) risk assessment model.

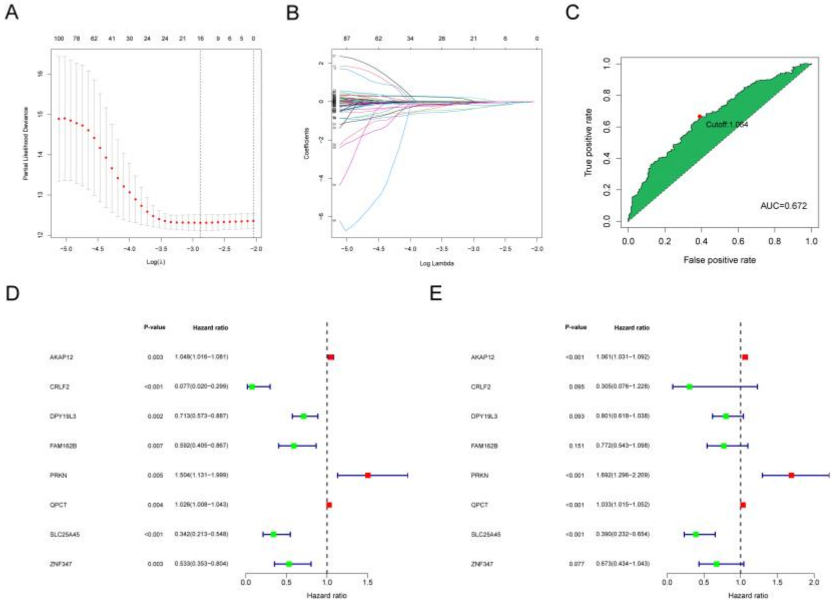

Figure S3. GEO database showed a strong association of FRZB with the immune checkpoints.

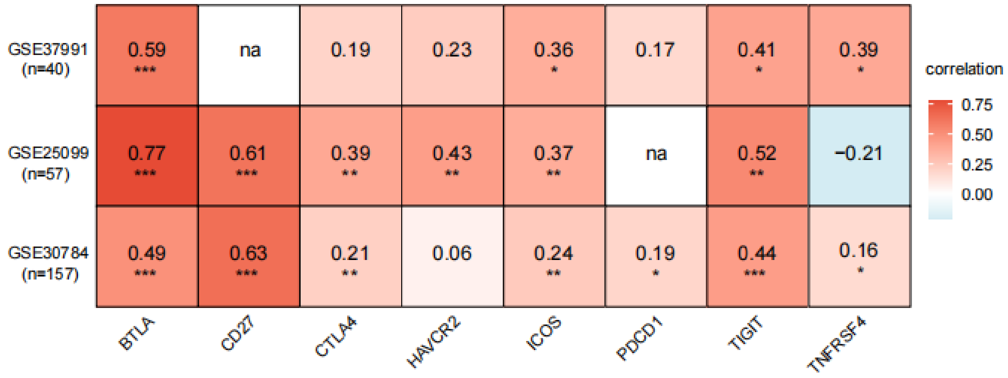

Supplement: Supplementary file 1 [file 1414-431X-bjmbr-57-e13368-suppl.zip › 1414-431X-bjmbr-57-e13368-supp_figures.pdf]
